# Arginase deficiency in Mexico: Insights from the experience of a metabolic reference center

**DOI:** 10.1016/j.ymgmr.2025.101238

**Published:** 2025-06-27

**Authors:** M. Vela-Amieva, C. Fernández-Lainez, S. Guillén-López, L. López-Mejía, M.A. Alcántara-Ortigoza, A. González del Angel, L. Fernández-Hernández, M.E. Reyna-Fabián, B. Estandía-Ortega, I. Ibarra-González, S.W. Ryu, H. Lee, on behalf of Rare Diseases Mexican Effort Group (RaDiMEG)

**Affiliations:** a Laboratorio de Errores Innatos del Metabolismo y Tamiz, Instituto Nacional de Pediatría, Mexico; b Facultad de Ciencias, UNAM, Investigación Científica C.U., Alc. Coyoacán, C.P. 04510, Cuidad de México, Mexico; c Laboratorio de Biología Molecular, Instituto Nacional de Pediatría, Av. Insurgentes Sur 3700C, col. Insurgentes Cuicuilco, C.P. 04530, Cuidad de México, Mexico; d Unidad de Genética de la Nutrición, Instituto de Investigaciones Biomédicas, UNAM, Av. Imán No. 1, col. Insurgentes Cuicuilco, C.P. 04530, Ciudad de México, Mexico; e 3billion, Inc., Seoul 06160, Republic of Korea

**Keywords:** Rare diseases, Urea cycle disorders, Hyperammonemia, Newborn screening

## Abstract

Arginase deficiency (ARG1d) is an inborn error of metabolism caused by pathogenic variants in *ARG1* gene, which causes a defective hydrolysis of arginine (Arg) to urea and ornithine. The molecular landscape of ARG1d in Mexico is poorly known. In this study, we present for the first time the clinical and genotypic overview of the largest cohort of ARG1d in Mexico. A retrospective analysis of the medical records of 24 ARG1d patients from a historical cohort of individuals with inborn errors of intermediary metabolism (1994–2024) from the National Institute of Pediatrics in Mexico City, was performed. Clinical, demographical, biochemical, anthropometric and molecular data were investigated in two moments, at diagnosis and at the last follow-up evaluation. It was found that only 7/24 patients were diagnosed by newborn screening (NBS). Additionally, we highlight the presence of the Portuguese NM_000045.4(ARG1):c.61C>T or p.(Arg21*) variant as the most frequent cause of ARG1d in Mexico, carried by 27.7 % of the patients. Our findings emphasize the debilitating and progressive nature of ARG1d, the prolonged diagnostic odyssey experienced by the patients (6.7 years), and the importance of training healthcare professionals to recognize the clinical features suggestive of the disease. We also underscore the critical need to advance early detection through expanded NBS in our country, as the early-diagnosed patients exhibited less severe outcomes and improved nutritional status compared to late-diagnosed ones. It was also noted that Mexican ARG1d patients have significant difficulties adhering to current nutritional treatment, and access to ammonium scavengers, thus other therapeutic options could be desirable.

## 1 Introduction

Arginase deficiency (ARG1d) (OMIM #207800, ORPHA:90; ICD-10:E72.2; ICD-11: 5C50.A2;) is an autosomal recessive inborn error of metabolism caused by a defect in the hydrolysis of arginine (Arg) to urea and ornithine. This is the final step of the urea cycle, which protects against excess ammonia [1]. Although ARG1d is a rare disorder, it has a high associated burden for affected families and healthcare systems, as it is a progressive condition that causes severe disability [2].

Arginase 1 (Arg1, EC 3.5.3.1) is encoded by the *ARG1* gene (6q23.2, MIM *608313), which consists of eight exons [3]. Currently, 137 pathogenic/likely pathogenic ARG1d-associated variants have been described in ClinVar (https://www.ncbi.nlm.nih.gov/clinvar/; accessed on December 3rd, 2024), and more than 200 have been described in the Leiden Open Variation Database v.3.0 [3] (LOVD, https://www.lovd.nl/; accessed on November 6th, 2024); however, a recent study by Cameron et al. analyzed 302 unpublished *ARG1* variants from gnomAD [4].

The Arg1 enzyme contains 322 amino acids [5] (https://www.uniprot.org/uniprotkb/P05089/entry). The functional unit is formed by a trimer consisting of three identical monomers of 35 kDa with a spherical structure of approximately 40x50x50 Å. This trimer requires a spin-coupled manganese cluster for its catalytic activity [1,6,7]. Each subunit contains an active site [7]. Dysfunction or absence of Arg1 activity leads to elevated blood concentrations of arginine, which is called hyperargininemia or argininemia [8]; additionally, di-aminoaciduria (elevated arginine, ornithine, and lysine in the urine) is a biochemical characteristic of the disease [9]. High blood levels of arginine in individuals with ARG1d have also been associated with changes in other amino acids, specifically citrulline, alanine, ornithine, glutamine, and asparagine [10].

The clinical features of patients with ARG1d are a reduction in linear growth, recurrent vomiting, feeding refusal, protein aversion, and anorexia [9,11,12]. Neurologic manifestations include intellectual disability, seizures, moderate intermittent episodes of hyperammonemia, and motor skills impairments such as abnormal gait, lower limb spasticity, and progressive spastic quadriplegia. Other manifestations, such as attention deficit hyperactivity disorder, aggressive behavior, and irritability, have been described [9,12]. In some cases, hepatomegaly and mild to severe liver dysfunction (elevated liver enzymes, clotting abnormalities, and cirrhosis) can occur [11].

The most common treatment for ARG1d patients is nutritional therapy. It comprises the restriction of intact protein, supplemented with a special formula for urea cycle disorders and nitrogen-scavenging drugs [9]. Liver transplantation has also been performed in some patients [13]. As a treatment goal, Arg blood concentrations are recommended to be as close to normal as possible [14]. However, this goal is challenging to accomplish since it has been demonstrated that Arg blood concentrations remain high in nutritionally treated patients [10], indicating the need for new therapeutic options such as enzymatic substitution [15].

Many countries offer early detection of ARG1d as part of the newborn screening (NBS) system. This method uses high arginine concentrations in blood spots and Arg/(Phe ^x^ Leu) and Arg/Orn ratios, which are analyzed through tandem mass spectrometry [16].

ARG1d is considered the most uncommon urea cycle disorder [12]. Using genetic databases, Catsburg et al. estimated the ARG1d global birth prevalence, resulting in 2.8 cases per million live births, ranging from 0.92 to 17.5 per million [17]. Interestingly, these same authors concluded that in Mexico, the ARG1d carrier frequency could be 0.0016, with an adjusted birth prevalence of 2.85 per million live births (95 %CI 1.99–4.20), which is similar to that estimated worldwide [17]. In fact, the birth prevalence of ARG1d in California, United States, has been estimated at 0.1 per 100,000 newborns (1:1,000,000) in nearly 3 million screened newborns, but, interestingly, the observed frequency of ARG1d in Hispanic newborns from California has been estimated to be 0.2 per 100,000 individuals or 1:500,000 [18]. Unfortunately, ARG1d epidemiology is unknown in Mexico, primarily because this disease is not included in the national mandatory NBS program. Only sporadic ARG1d case reports have been published in Mexico [19] and in other countries, without any description of the specific geographic origin of the affected patients [20]. Our group has published case series reports of several ARG1d patients, but only as part of other cohort descriptions [21,22]. This study aimed to provide insights into Mexican patients with ARG1d, including their biochemical, clinical, and demographic characteristics, diagnostic odysseys, adherence to nutritional treatment, and genotypic landscape.

## 2 Methods

### 2.1 Patients

This study involved a retrospective analysis of the medical records of patients diagnosed with ARG1d from a historical cohort of individuals with inborn errors of intermediary metabolism (IEiM) (1994–2024) of the National Institute of Pediatrics (*Instituto Nacional de Pediatría*), which is a tertiary specialized referral center from the Ministry of Health in Mexico City. All patients underwent a comprehensive clinical evaluation before their inclusion in this study. Human phenotype ontology (HPO) terms were used to categorize the clinical manifestations (https://hpo.jax.org/) (Supplementary Table S1). Patients were classified according to their moment of diagnosis as early diagnosed, those who were detected by newborn screening in the first month of life, and as late diagnosed those diagnosed out of the newborn period, who already presented any sign of the disease. Biochemical, demographic, and genotypic data were collected at two times: at time of diagnosis and at the time of the last follow-up evaluation.

All patients underwent nutritional treatment consisting of a low-protein diet supplemented with an essential amino acid formula to achieve their energy and protein requirements according to age. Sodium benzoate (250–500 mg/kg/d), was used as a nitrogen scavenger [23,24].

Treatment adherence was measured using a customized scale adapted from the Morisky-Green medication adherence scale. Adherence was also measured using a 3-day dietary recall and categorized as good when the medical and nutritional recommendations were followed strictly more than 80 % of the time, regular when the recommendations were followed between 50 and 80 % of the time, and bad when the recommendations were followed less than 50 % of the time [25].

To evaluate linear growth, the height/age *Z*-score and body mass index (BMI), and Z-score were calculated with Anthro® (Version 3.2.2.1, Geneva, Switzerland) for patients aged 0 to 5 years and AnthroPlus® software (Version 1.0.4, Geneva, Switzerland) for patients aged 5 to 18 years. Stunting was defined by a height for age Z-score less −2 standard deviations (SDs), on the basis of the World Health Organization (WHO) definition [26].

### 2.2 Diagnostic odyssey and mortality analyses

The diagnostic odyssey was defined as the time between symptom onset and the final diagnosis, in years. The mortality rate was estimated as the proportion of deceased ARG1d subjects from the total cohort.

### 2.3 Amino acid and ammonia blood analyses

Amino acid blood concentrations were quantified through high-performance liquid chromatography or tandem mass spectrometry following previously reported methodologies [27]. Ammonia levels were determined in our institution's central laboratory, with normal reference values ranging from 9 to 35 μmol/L.

To explore the utility of arginine ratios as disease biomarkers, a metabolomic analysis was performed using MetaboAnalyst version 6.0, a comprehensive free web-based tool for metabolomics data processing [28]. The ratios of amino acids to acylcarnitines were analyzed and compared with those of historical laboratory normal controls.

### 2.4 Whole exome sequencing (WES)

For index cases (*N* = 9) and a few affected relatives (*N* = 4), genomic DNA was extracted from peripheral blood leukocytes or dried blood spot (DBS) cards using standard protocol (QIAamp DNA Blood Mini Kit, Hilden, Germany), for peripheral blood collected in EDTA tubes and AccuBuccal DNA Preparation Kit (AccuGene, Seoul, South Korea). Exome capture of the protein-coding region of approximately 20,000 known genes was performed using the xGen Exome Research Panel v2 alone or supplemented with the xGen human mtDNA Panel and the xGen Custom Hyb Panel v1 (Integrated DNA Technologies, Coralville, Iowa, USA). Sequencing was conducted on the Illumina NovaSeq 6000 platform (Illumina, San Diego, CA, USA). The sequencing reads were aligned to the GRCh37/hg19 human reference genome and the mitochondrial genome's Revised Cambridge Reference Sequence (rCRS). As previously described [29], variables were called using open-source bioinformatics tools and in-house software. This included the identification of single-nucleotide variants (SNVs), small insertions and deletions (INDELs), copy number variants (CNVs), and repeat expansion variants. Variant annotation, filtering, and classification were performed using the EVIDENCE platform [30]. Common variants with allele frequencies greater than 5 % in the gnomAD database or greater than 1 % in the internal database were filtered out, except for variants known to be pathogenic or likely pathogenic (P/LP). The pathogenicity of each variant was evaluated according to the American College of Medical Genetics and Genomics and the Association for Molecular Pathology (ACMG/AMP) guidelines [31], supplemented by a quantitative Bayesian scoring system [32].

### 2.5 Statistical analyses

The distribution of the data was calculated using the Shapiro–Wilk test. One-way ANOVA followed by Dunn's multiple comparisons adjustment was used to analyze the normally distributed data. Nonparametrically distributed data were analyzed using the Mann–Whitney *U* test and Dunn's multiple comparisons adjustment test. The results are expressed as the means ± SDs or medians and interquartile ranges (IQRs), for data with parametric and nonparametric distributions, respectively. A *p*-value of <0.05 was considered to indicate statistical significance (* *p* < 0.05; ** *p* < 0.01; *** *p* < 0.001; **** *p* < 0.0001). For the analysis of categorical variables, Fisher's exact test was performed. All these statistical analyses were performed with GraphPad Prism version 10.4.1. (532) software.

### 2.6 Ethical considerations

The Research, Ethics, and Biosafety Institutional Committees approved the protocol (institutional number 2022/051). All the studies and procedures followed the ethical principles of the actualized Helsinki Declaration [33]. Written informed consent was obtained from the parents of patients who underwent WES, including authorization for the investigation of incidental and secondary findings, as well as for being informed about them.

## 3 Results

Since 1994, 21 unrelated families with ARG1d have been diagnosed at our center (24 patients, 15 males and nine females). Two and three affected siblings were registered in two and one families, respectively. Consanguinity was documented in 3 out of 21 families (14.2 %). Endogamy was not recorded. Among the 21 families, 14 (66.6 %) came from Mexico City and its surrounding state (Mexico State), whereas the remaining (*N* = 7, 33.3 %) came from the southeastern region of Mexico (Chiapas and Tabasco States). Of the 24 included ARG1d patients, 17 had clinical follow-up until at least the age of 20 years. Only 7 out of 24 (29.2 %) patients were detected by NBS, while the remaining 17 were diagnosed with clinical suspicion. The mortality rate reported in this study was 16.6 % (*N* = 4/24 patients, 16 per 10,000), and the median age at death was 14.8 years.

The mean age at diagnosis of the seven patients detected by NBS was 26.7 days (minimum of one day and maximum of 3 months), whereas in those diagnosed later by clinical suspicion, the mean age at diagnosis was 8.8 years (3.9 months to 20 years), with a mean age at the onset of symptoms of 2.1 years. The average diagnostic odyssey of the patients was 6.7 years (minimum of one month to a maximum of 16.3 years). Interestingly, the diagnostic odyssey included, on average, visits to up to four different pediatric specialists, mainly orthopedists, physical rehabilitation staff, neurologists, and geneticists; however, visits to neurologists and geneticists were the most effective, since they were the medical specialists that mainly suspected the disease and performed the reference to our center.

**Fig. 1:**
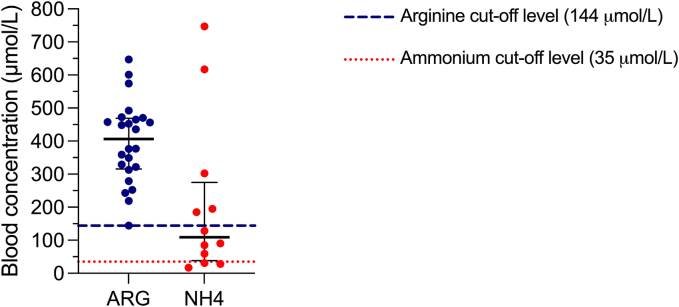
Biochemical characteristics in ARG1d Mexican patients at the moment of diagnosis. Data is presented as median with interquartile range. ARG: Arginine, NH4: ammonium.

At diagnosis, the Arg blood concentrations of the ARG1d patients, with and without NBS detection, were 397.2 μmol/L ± 123.5, which were all above the cutoff value (144 μmol/L). The mean blood ammonium concentration was 207 μmol/L (minimum 17 μmol/L, maximum 747 μmol/L, reference value <35 μmol/L); however, it was within normal ranges in three patients (Fig. 1).

**Fig. 2:**
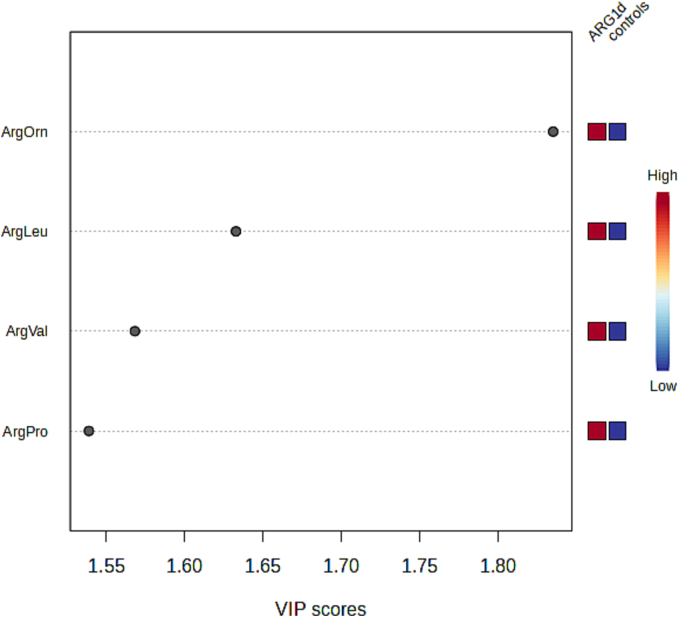
Metabolomic analyses of ARG1d patients (*n* = 15) at diagnosis, compared to laboratory historical controls (*n* = 389). PLSD graph of ARG1d patients compared with historical controls.

The metabolomic analysis revealed that the amino acids arginine, ornithine, citrulline, leucine, proline, valine, glycine, tyrosine, and phenylalanine, along with isovalerylcarnitine (C5) and acetylcarnitine (C2), were key contributors to the differences observed between patients and our normal historical controls. Consequently, a second metabolomic analysis was conducted focusing on the ratios of arginine to these amino acids and acylcarnitines. These arginine-based ratios demonstrated high discriminative power, with variable importance in project (VIP) scores greater than 1.5; Arg/Orn, Arg/Val, and Arg/Leu were the ratios with the highest VIP scores (Fig. 2).

### 3.1 Clinical manifestations

**Fig. 3:**
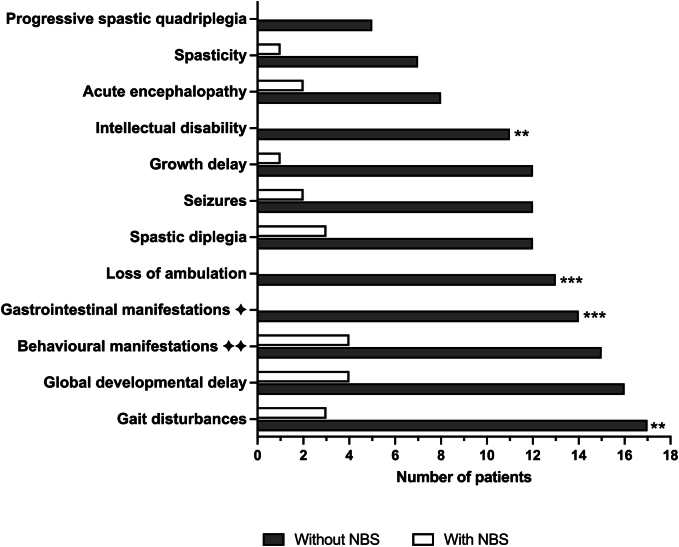
Clinical manifestations of Mexican ARG1d patients, compared by type of disease detection. ** *p* < 0.01; *** *p* < 0.0001. The HP number of each clinical manifestation can be consulted in the supplementary table S1.  Includes vomiting, feeding difficulties in infancy, protein avoidance, and anorexia.  Includes atypical behavior, psychiatric disturbances, attention deficit hyperactivity disorder, aggressive behavior, and irritability.

In late-diagnosed ARG1d patients, gait disturbances and global developmental delay were the most frequent manifestations with 100 % and 94 %, respectively; however, in early NBS-detected ARG1d patients, behavioral manifestations and global developmental delay were observed most frequently both with 57.1 %, but in a significantly lower proportion than in late-detected patients. Gait disturbances, gastrointestinal manifestations, loss of ambulation, and intellectual disability were substantially lower in the NBS group than in the late diagnosis group (Fig. 3).

Only 17/24 patients followed nutritional treatment, 3/7 from the NBS group, and 14/17 from the late-diagnosed group. Treatment adherence was considered good in 11.8 %, regular in 11.8 %, and poor in 76.4 % of the patients. Their most recent nutritional assessment revealed that in the late-diagnosed group, only 2/14 (14.2 %) had appropriate growth for age, whereas 2/3 (66.7 %) of early NBS-diagnosed patients had appropriate growth for age (Table 1).

**Table 1:** Nutritional assessments and treatment adherence of Mexican ARG-1 patients, considering the type of diagnosis of the disease.

Patient ID	Age at assessment (years)	Height/age *Z*-score (−2 − +2)⁎	BMI Z-score (−2 − +1)⁎	Nutritional diagnosis	Treatment adherence
Patients diagnosed by NBS					
Arg-23	0.34	−1.36	−0.13	Appropriate growth for age	Good
Arg-24	18	−1.29	−0.87	Appropriate growth for age	Good
Arg-16	8.3	−2.99	−1	Stunted	Poor
Late diagnosed patients					
Arg-12	5.8	−1.17	−0.65	Appropriate growth for age	Regular
Arg-03	6.7	−1.9	0.61	Appropriate growth for age	Regular
Arg-04	6.4	−2.73	0.33	Stunted	Poor
Arg-09	16.8	−2.87	−4.29	Stunted and severe malnutrition	Poor
Arg-18	10.5	−2.9	−0.51	Stunted	Poor
Arg-10	18	−3	−0.04	Stunted	Poor
Arg-07	3.8	−3.34	1.93	Stunted	Poor
Arg-17	15.4	−3.62	−1.82	Stunted	Poor
Arg-02	11.2	−3.7	−1.44	Stunted	Poor
Arg-22	17.3	−3.78	1.33	Stunted and overweight	Poor
Arg-15	12.8	−3.85	−1.01	Stunted	Poor
Arg-01	18	−4.7	−5.84	Stunted and severe malnutrition	Poor
Arg-13	17.6	−4.83	−3.52	Stunted and severe malnutrition	Poor
Arg-06	15.1	−6.29	−3.21	Stunted and severe malnutrition	Poor

⁎ The reference values are according to WHO 2007 standards [26].

### 3.2 Spectrum of *ARG1* variants

Thirteen patients from nine families underwent molecular testing through WES. Nine different pathogenic variants were identified in *ARG1* (Table 2). Neither incidental nor secondary findings were documented. The most prevalent variant was the nonsense NM_000045.4(ARG1):c.61C>T or p.(Arg21*) variant [dbSNP: rs104893944; ClinVar: 2397], which was found at the frequency of 5/18 alleles (27.7 %). It constituted the homozygous genotype of one patient (Arg-03) and the compound heterozygous genotype of other two unrelated families (Table 3). The NM_000045.4(ARG1):c.425G>A or p.(Gly142Glu) [dbSNP: rs767219084; ClinVar: 556022] variant was found in 4/18 alleles (22.3 %), and the NM_000045.4(ARG1):c.466-1G>C or p.? [dbSNP: rs1064794165; ClinVar: 419876] variant that was found in 3/18 alleles (16.7 %). No uncertain significance (VUS) or benign variants were found, although only the in-frame microdeletion NM_000045.4(ARG1):c.767_769del p.(Glu256del), considered a VUS in the main genotypic databases [dbSNP: rs1554251236, ClinVar: 551012], was reclassified as pathogenic according to the ACMG/AMP pathogenicity variant classification criteria [31,32] (Table 2).

**Table 2:** Identified *ARG1* variants and their allelic frequencies in the studied Mexican ARG1d patients.

Variant number	c.DNA NM_000045.4	Protein change	Exon	Type	Pathogenicity	# alleles (*N* = 18) (frequency %)
1	c.61C>T	p.(Arg21*)	2	Nonsense	Pathogenic	5 (27.7)
2	c.425G>A	p.(Gly142Glu)	4	Missense	Pathogenic (PP3, PP5, PM1, PM2, PM3)	4 (22.3)
3	c.466-1G>C	p.(?)	Splice site	Splicing defect	Pathogenic	3 (16.7)
4	c.3G>A	p.(Met1?)	1	Missense	Pathogenic	1 (5.55)
5	c.223A>T	p.(Lys75*)	3	Nonsense	Pathogenic	1 (5.55)
6	c.767_769del	p.(Glu256del)	7	In-frame and frameshift indels	Pathogenic (PS4, PM1, PM2, PM4, PP3, PP4)	1 (5.55)
7	c.787G>T	p.(Glu263*)	7	Nonsense	Pathogenic	1 (5.55)
8	c.871C>T	p.(Arg291*)	8	Nonsense	Pathogenic	1 (5.55)
9	c.892G>C	p.(Ala298Pro)	8	Missense	Pathogenic (PP5, PM5, PP3, PM1, PM2, PM3)	1 (5.55)

**Table 3:** Genotype/phenotype correlation, adherence to treatment and outcome of ARG1d Mexican patients.

Patient ID	Genotype c.DNA	Genotype (protein)	Familial (F) Unique (U)	Type of detection	Expected phenotype	Nutritional treatment start	Adherence to treatment	Observed outcome
Arg-04	c.61C>T(;)466-1G>C	p.Arg21*(;)?	U	NBS	Severe	Early	Poor	Alive with mild global neurodevelopmental delay
Arg-24			U	NBS	Severe	Early	Good	Alive with age-appropriate physical, intellectual and educational development
Arg-03	c.61C>T(;)61C>T	p.Arg21*(;)Arg21*	U	Late	Severe	Late	Regular	Death
Arg-12	c.61C>T(;)892G>C	p.Arg21*(;)Ala298Pro	F (2 siblings)	Late	Severe	Late	Regular	Alive with severe clinical presentation
Arg-13							Poor	
Arg-01	c.425G>A(;)871C>T	p.Gly142Glu(;)Arg291*	F (2 siblings)	Late	Severe	Late	Poor	Alive with severe clinical presentation
Arg-02								
Arg-06	c.3G>A(;)767_769del	p.Met1?(;)Glu256del	U	Late	Severe	Late	Poor	Alive with severe clinical presentation
Arg-10	c.425G>A(;)425G>A	p.Gly142Glu(;)Gly142Glu	U	Late	Severe	Late	Poor	
Arg-09	c.223 A >T(;)425G>A	p.Lys75*(;)Gly142Glu	U	NBS	Severe	Early	Poor	Alive with mild global neurodevelopmental delay
Arg-16	c.466-1G>C(;)787G>T	p.?(;)Glu263*	F (3 siblings)	NBS	Severe	Early	Poor	Alive with severe clinical presentation
Arg-17				Late	Severe	Late	Poor	Death
Arg-18				Late	Severe	Late	Poor	Alive with severe clinical presentation

Eight different biallelic *ARG1* genotypes were found. An exploratory genotype/phenotype correlation is shown in Table 3, highlighting the moment of treatment initiation and compliance. According to the pathogenicity classification of the variants, the expected phenotype of all the genotypes was severe. This was true for all the patients, except for patient Arg-24 who had good medical outcomes associated with early diagnosis and treatment, as well as good compliance with nutritional treatment (Table 3).

## 4 Discussion

Herein, we present for the first time a clinical and genotypic overview of ARG1d in a cohort of Mexican patients. The findings of this study highlight the debilitating and progressive nature of ARG1d, the prolonged diagnostic odyssey experienced by patients not diagnosed via NBS (averaging 6.7 years), and the importance of training health care professionals, particularly orthopedists, pediatricians, and rehabilitation specialists, to recognize the clinical features suggestive of the disease. All these findings underscore the critical need to advance early detection through expanded NBS in our country. As described herein, early diagnosed patients exhibit less severe outcomes and improved nutritional status than late-diagnosed patients do. The fact that individuals treated from birth appear to have minimal symptoms has been described by other authors [34]. Nevertheless, it is essential to note that treatment adherence is fundamental for a good outcome in addition to early detection (Table 1).

Biochemically, all patients presented the typical amino acid profile of ARG1d, characterized by high levels of arginine (Fig. 1), as well as alterations in the well-documented Arg/Orn ratio [35]. By the metabolomic analysis we corroborated the value as biomarkers of Arg/Orn Arg/Phe, Arg/Phe*Leu [16], remarkably we also found that the ratios Arg/Val and Arg/Leu had a good discriminative power as ARG1d biomarkers (Fig. 2), however, these latter ratios deserve further exploration in bigger cohorts.

Unfortunately, poor adherence to treatment was recorded in our ARG1d patient cohort (76.4 %). This can be related to difficulties that patients face in following the diet, since it is very restrictive and time-consuming. Moreover, access to formulas is not always guaranteed for all of them. This situation highlights the need for other therapeutic options [15]. These issues in adherence to treatment may explain why some of our patients, despite early detection, exhibited significant manifestations of the disease, mainly behavioral problems, global developmental delay, gait disturbances, spastic diplegia, and seizures, among others (Fig. 3).

In this cohort, four patients with a median of 14.8 years, died. This finding is different from that reported in a systematic review of 157 ARG1d case reports, where a median of 5.7 years at death was identified [8], which could be related to our small number of patients analyzed. In our study, the principal causes of death were respiratory failure secondary to recurrent respiratory infections and inadequate management of airway secretions, accompanied by hyperammonemia crises and sepsis.

None of the patients detected early had intellectual disability, loss of ambulation, gastrointestinal manifestations, or quadriplegia (Fig. 3). Furthermore, 66.7 % of patients identified through NBS were classified as appropriate growth for age, whereas only 14.2 % of those diagnosed late were classified as individuals with appropriate growth for age (Table 1). The Arg-24 patient despite having a genotype composed of two severe and pathogenic variants, had the best outcome, as documented during his medical follow-up over 20 years (Table 3). He has no intellectual disability, spasticity, or other neurological symptoms, and currently, he is studying at a college. Thus, the combination of neonatal diagnosis, early treatment initiation, and strict adherence to dietary treatment seems to be pivotal factors in attenuating the disease phenotype.

As previously noted [12], gait disturbances progressing to spastic tetraplegia and global neurodevelopmental delay, were the most remarkable clinical manifestations in our cohort of Mexican patients (Fig. 3). Although hepatomegaly and liver dysfunction have been observed in a minority of ARG1d patients (13.6 %) [36], in the patient files of this cohort, no liver abnormalities were documented. In ARG1d liver abnormalities are poorly studied; thus, the underlaying mechanisms are not completely understood. However, Arg accumulation can increase oxidative stress. Another cause of liver damage could be reduced urea cycle activity, which could cause damage to liver cells via mitochondrial dysfunction [9,37]. In accordance with the natural history of ARG1d where few cases begin in the first days of life [12], only one patient (Arg-01, 4.16 %) presented with clinical manifestations during the newborn period.

Hyperammonemia in ARG1d patients is rare and moderate [3,11]. Nevertheless, in some cases, hyperammonemia can reach toxic concentrations that have resulted in coma. In the present study, ammonium blood concentrations ranged between 2 and 21 times above the reference value, and 83 % of our patients presented hyperammonemia at the time of diagnosis (Fig. 1). Hyperammonemia is commonly the first clinical data to leading to suspicion of the disease as part of the medical approach to rule out a urea cycle defect, and it must be treated promptly to avoid neurological sequelae.

Considering the low prevalence of the disease worldwide, we are aware of the high number of patients being followed in our center (*N* = 24) [12]. This fact supports the notion that ARG1d could be more frequent in individuals with Mexican ancestry, as previously suggested by several authors [17,18]. Furthermore, Bin Sawad et al., in a systematic review of 157 ARG1d patients, reported that the Hispanic/Latino ethnic group had the greatest number of affected subjects [36]. This compels us to investigate more about this disease further in Mexico, especially in southern Mexico, considering that close to 30 % of our patients came from that region.

With respect to the mutational *ARG1* spectrum, the c.61C>T or p.(Arg21*) was the most frequent identified variant (5/18 alleles, 27 %, 4 families). It was found in a compound heterozygous state in two siblings (Arg-12, Arg-13) and in two singleton cases (Arg-04, Arg-24), whereas only one patient was homozygous (Arg-03) (Table 3). It is important to note that this variant is not more frequently found in Admixed American population than in the rest of the world [38,39] Since the p.(Arg21*) is a null variant with an expected severe effect in the enzyme. Thus, the severe phenotype observed in our homozygous patient (Arg-03) supports the severity of the variant.

Despite being unrelated, the early NBS-detected patients Arg-04 and Arg-24 shared the same *ARG1* genotype, but their clinical outcomes were different, which agrees with that previously described for this pathogenic variant, which was firstly reported in 1999 in four p.(Arg21*) homozygous Portuguese patients [40]. The clinical presentation of these Portuguese patients was remarkably variable, manifesting as hepatic dysfunction and extrapyramidal irritative syndrome. Additionally, the youngest patient reported by these authors presented at one month of age with a cholestatic syndrome; others presented with symptoms between 2 and 5 years of age, with extrapyramidal irritative syndrome, liver dysfunction, and neurodevelopmental delay. The Arg blood levels registered in these patients were between 410 and 556 μmol/L, but patients with higher Arg concentrations of 1475 μmol/L also presented hyperammonemia and coma [40]. The c.61C>T or p.(Arg21*) *ARG1* variant has also been commonly reported in Brazil, yielding a heterogeneous clinical picture from moderate to severe manifestations even in homozygous p.(Arg21*) patients [41]. However, it should also be noted that environmental and familial factors and other modifiers of disease progression, such as adherence to treatment, could also significantly influence the course of the disease. The presence of this variant of Portuguese origin as the main genotypic etiology of ARG1d in this cohort of Mexican patients is striking. Although it is known that some individuals of Portuguese ancestry were present during the colonization of Mexico, this occurred in a much smaller proportion than in countries such as Brazil [42], thus a possible Portuguese founder effect for the c.61C>T or p.(Arg21*) variant deserves further research.

The phenotype observed in two compound heterozygous c.61C>T(;)466-1G>C unrelated patients diagnosed by NBS (Arg-04 and Arg-24), was discordant, as mild global neurodevelopmental delay was observed only in Arg-04, which may be related to the poor treatment adherence.

The second most frequently found variant c.425G>A or p.(Gly142Glu) was found in a compound heterozygous state in two siblings (Arg-01, Arg-02) and two unique cases (Arg-09, Arg-10). In the family consisting of Arg-01 and Arg-02 patients, the index patient was diagnosed at 5 years, whereas his sibling was diagnosed when he was 13 years old. Owing to these late diagnoses, both siblings presented severe manifestations. Interestingly, the first ARG1d patient reported to carry this pathogenic variant was a Hispanic boy from California, USA, who was detected by NBS, with good outcomes and normal neurodevelopment [43].

The c.466-1G> C (formerly IVS4-1G>C) or p.(?) variant was found in a family with three affected children (Arg 16, Arg 17, and Arg 18), that came from the southern state of Tabasco. The affected members were diagnosed at 8 years (Arg-17), 2.5 years (Arg-18), and at birth (Arg-16). The youngest child, despite being diagnosed early through NBS, experienced seizure episodes. Importantly, all affected children in Family 3 had very poor adherence to treatment, mainly due to difficulties in obtaining the prescribed medical formula. This variant was previously identified in an affected ARG1d1 newborn from Yucatan State which shares a border with Tabasco State [19]. Thus, we highlight that research on ARG1d in Southeastern Mexico must be performed in detail in further studies, and that pediatricians in that area must be especially alert and trained in the suspicion and diagnosis of this disease.

The in-frame microdeletion c.767_769del p.(Glu256del), which is considered a VUS in ClinVar (ID: 551012) was identified alongside the pathogenic start-lost variant p.(Met1?) in patient Arg-06. This variant affects the “Arginase-like; Arginase types I and II and arginase-like family” functional domain (a.a. 9 to 303; https://www.ncbi.nlm.nih.gov/Structure/cdd/cddsrv.cgi?uid=212536). Moreover, the E256 position is highly conserved across species, from yeast to humans, and this amino acid is located inside of an allosteric site and magnesium-binding region. We re-classified it as pathogenic, since it complies with the PS4, PM1, PM2, PM4, and PP3 (CADD score of 20.9 and MutPred-Indel indicates a deleterious effect), and PP4 ACMG/AMP pathogenicity criteria [31,32].

While our study has several limitations, including incomplete clinical data, especially that related to a comprehensive liver function evaluation which is a key feature of ARG1d patients [10] and loss of medical follow-up for some patients, as well as the small number of included patients, with unavailability of all *ARG1* genotypes, our study clearly indicates that ARG1d is present in Mexico and affects patients who are not detected and treated early and who do not adhere to treatment, consequently suffering devastating consequences that lead to severe intellectual and motor disability, and eventually death. Unfortunately, we also noted that diagnosed Mexican patients have significant difficulties adhering to the recommended nutritional treatment, so access to other therapeutic options could be desirable. Similarly, we highlight the presence of the Portuguese c.61C>T or p.(Arg21*) variant as a cause of ARG1d in Mexico. Our findings could justify the incorporation of the early detection of ARG1d into the mandatory NBS program in Mexico, as well as the development of programs to train health personnel to suspect the disease and provide better medical follow-up of affected patients.

## CRediT authorship contribution statement

**M. Vela-Amieva:** Writing – review & editing, Writing – original draft, Visualization, Validation, Software, Resources, Methodology, Investigation, Funding acquisition, Formal analysis, Data curation, Conceptualization. **C. Fernández-Lainez:** Writing – review & editing, Writing – original draft, Visualization, Supervision, Software, Resources, Project administration, Methodology, Investigation, Funding acquisition, Formal analysis, Data curation, Conceptualization. **S. Guillén-López:** Writing – review & editing, Methodology, Investigation, Formal analysis. **L. López-Mejía:** Writing – review & editing, Methodology, Investigation, Formal analysis. **M.A. Alcántara-Ortigoza:** Writing – review & editing, Writing – original draft, Visualization, Validation, Resources, Investigation, Formal analysis, Data curation. **A. González del Angel:** Writing – review & editing, Validation, Methodology, Formal analysis. **L. Fernández-Hernández:** Writing – review & editing, Validation, Methodology, Formal analysis, Data curation. **M.E. Reyna-Fabián:** Writing – review & editing, Validation, Methodology, Formal analysis, Data curation. **B. Estandía-Ortega:** Writing – review & editing, Visualization, Investigation, Formal analysis. **I. Ibarra-González:** Writing – review & editing, Methodology. **S.W. Ryu:** Writing – review & editing, Software, Methodology. **H. Lee:** Writing – review & editing, Software, Methodology.

## Institutional review board statement

This study was conducted according to the guidelines of the Declaration of Helsinki and approved by the Institutional Review Board (Ethics, Research, and Biosafety Committees) of the National Institute of Pediatrics (Reference protocol numbers 052/2016 and 2024/032).

## Funding

This research was funded by the Instituto Nacional de Pediatría, Secretaría de Salud (Recursos Fiscales 2022–2024, Programa E022 Investigación y Desarrollo Tecnológico en Salud, Ciudad de México, México, protocol numbers 052/2016 and 2024/032). WES studies were funded by a 3Billion grant to M.V.-A.

## Declaration of competing interest

None.

## Data Availability

Data will be made available on request.

## Appendix A Supplementary data

Supplementary material Image 3
